# Evaluating the cardioprotective effect of metformin on myocardial ischemia–reperfusion injury using dynamic ^18^F-FDG micro-PET/CT imaging

**DOI:** 10.1186/s12872-022-02750-2

**Published:** 2022-07-10

**Authors:** Hang Su, Diyu Lu, Mingkui Shen, Li Feng, Chuangye Xu

**Affiliations:** 1 Department of Nuclear Medicine, The Central Hospital of Wuhan, Tongji Medical College, Huazhong University of Science and Technology, Wuhan, People’s Republic of China; 2 School of Medicine, Southern University of Science and Technology, 1088 Xueyuan Avenue, Nanshan District, Shenzhen, 518055 People’s Republic of China

**Keywords:** PET/CT, ^18^F-FDG, Myocardial ischemia–reperfusion injury (MIRI), Metformin, Cardioprotective effect

## Abstract

**Background:**

The molecular mechanisms of protective effect of metformin (Met) on ischemic myocardium have not been fully understood. This study aims to evaluate the cardioprotective effect of metformin on myocardial ischemia–reperfusion injury (MIRI) in rat models at different time points using dynamic ^18^F-FDG micro-PET/CT imaging.

**Methods:**

The I/R injury model in SD rats was established by ligation of left anterior descending coronary artery near the pulmonary arch root for 30 min. SD rats (n = 12) were randomly divided into 2 groups: Control group (n = 6) without any intervention and Met group (n = 6) with oral administration of metformin (50 mg/kg) twice a day. Gated ^18^F-FDG (40Mbq) micro-PET/CT imaging was performed for 10 min at different time points (day 1st, day 7th, day 14th and day 30th after operation). Volumes of interest were drawn to identify different myocardium regions (ischemia center, peri-ischemia area and remote area). Standardized uptake values (SUVs) (SUV_mean_ and SUV_max_) were analyzed to evaluate the FDG uptake activity, and then the center/remote ratio was calculated. In addition, the left ventricular (LV) end-diastolic volume (EDV), end-systolic volume (ESV) and LV ejection fraction (LVEF) were obtained. On the 30th day, all rats were scarified and myocardial ischemia was analyzed by HE staining and confirmed by pathology.

**Results:**

In the Control group, the center/remote ratio showed no obvious change trend at each time point after reperfusion, while the LV EDV increased gradually over time, and they were significantly negatively correlated (r = − 0.507, *p* < 0.05). In the Met group, the center/remote ratio gradually increased with time, there was no significant correlation between center/remote ratio and LV EDV (r = − 0.078, *p* > 0.05). On the 30th day, the center/remote ratio of the Met group was significantly higher than that of the Control group (0.81 ± 0.06 vs. 0.65 ± 0.09, *p* < 0.05), while LV EDV in Met group was significantly lower than in Control group (358.21 ± 22.62 vs. 457.53 ± 29.91, *p* < 0.05). There was no significant difference of LVEF between Met group and Control group at different time points after reperfusion (*p* < 0.05). HE staining showed that the myocardial infarction and fibrosis in ischemic center area of the Control group was more serious than that of the Met group.

**Conclusions:**

Met could attenuate the severity of MIRI, delay and prevent the progress of LV remodeling. The cardioprotective progress could be dynamically assessed by ^18^F-FDG micro-PET/CT imaging.

## Background

Myocardial infarction (MI) is one of the major cardiovascular diseases that seriously endanger human health [1]. Early reperfusion after primary percutaneous coronary intervention (PCI) is still the standard and most effective method to treat myocardial infarction MI [2, 3]. However, reperfusion therapy is a "double-edged sword", which can not only restore the supply of myocardial oxygen, but also aggravate the secondary damage of ultrastructure, metabolic level, cell activity and local function of myocardial cells, leading to an irreversible myocardial ischemia–reperfusion injury (MIRI) [4–6]. This undesirable MIRI can lead to myocardial remodeling, myocardial fibrosis and eventually cardiomyocyte death [7–10], thus causing severe systolic dysfunction. Although the treatment of MIRI has made remarkable progress in recent decades, the morbidity (up to 25%) and mortality of heart failure after acute MI are still at a high level, and the quality of life and prognosis are poor [11, 12]. Therefore, in the early stage of myocardial ischemia, targeted diagnosis and treatment for MIRI can prevent its occurrence and development, which is of great significance to improve left ventricular function and long-term prognosis of MI patients.

As a commonly used hypoglycemic agent for patients with type 2 diabetes mellitus (T2DM), the protective effect of metformin (Met) on ischemic myocardium has also attracted much attention [13–16], but its molecular mechanisms have not yet been fully understood. UKPDS (United Kingdom Prospective Diabetes Study) [17] showed that the improvement of blood glucose control in overweight patients treated with metformin could reduce cardiovascular end points related to diabetes compared to conventional methods. In few previous studies, MIRI models of mice and rats in vivo and in vitro were used to evaluate the cardiovascular protective effect of Met on metabolic parameters and left ventricular (LV) function [18–20]. However, only pathological and immunohistochemical methods are adopted to evaluate the efficacy of Met at the endpoint of treatment, lacking systematic and continuous specific molecular imaging information in vivo, including myocardial cell perfusion, cell activity, energy metabolism, inflammatory response and dynamic changes of neuronal activity. Therefore, the dosing regimen (dose and time) of Met for MIRI has not yet been determined, and the dynamic effect of Met MIRI pathophysiology is still unclear.

Positron emission tomography/computed tomography (PET/CT) is a highly sensitive and non-invasive molecular imaging modality which provides an opportunity to explore various pathophysiological processes within MI in vivo by using various radiotracers [21, 22]. It could provide quantitative and semi-quantitative information of local anatomical morphology, metabolism and functional parameters [23]. Therefore, it may be used to dynamically and real-timely evaluate the pathophysiological characteristics of MIRI in vivo at the molecular level, including blood perfusion, cardiomyocyte metabolism and cell activity. Because the size of rat can provide a more detailed image in vivo and the characteristics of myocardium metabolism in rats are more similar to those of humans, Sprague–Dawley (SD) rat model is becoming attractive in cardiovascular system PET/CT imaging. However, there is little research about MIRI in the SD rat model.

In this study, ^18^F-FDG PET/CT myocardial metabolism imaging would be used in the SD rat model to dynamically evaluate the effects of Met intervention on MIRI at molecular level.

## Methods

Male SD rats (4–6 weeks) were purchased from SPF (Beijing) Biotechnology Co., LTD. Rats were maintained in a temperature-controlled room (25 °C) with a natural day/night cycle and fed with a standard rodent diet and water. Photographic developer ^18^F-FDG was purchased from HTA Co., Ltd. (Beijing, China). The experimental apparatuses mainly included: Inveon Micro-PET/CT scanner (SIEMENS, Germany), Matrx VMR anesthesia machine (Matrx, USA), LeicaRM2235 slicer (Beijing light division technology development Co., Ltd.) and immunohistochemical microscope imaging system (ECLIPSE 50I/55I, NICON). The experiments were carried out under the permission of the Project (NO.: AEEI-2019-167) approved by the Animal Care Committee of Capital Medical University, in compliance with the Animal Management Rule of the Chinese Ministry of Health (documentation 55, 2001) for the care and use of animals.

### Establishment of rat MIRI models

All rats were anesthetized by intravenous injection of 10% chloral hydrate, and then mechanically ventilated by tracheal intubation. The left anterior descending (LAD) coronary artery was ligated near the root of lung arch for 30 min, and the ischemia–reperfusion injury model was established. Changing gray of myocardium indicated success of ligation. When the color of myocardium from the ligation area returned to normal, it meant the reperfusion was successful.

### Animal groups

20 rats were randomly divided into two groups: (1) Control group (n = 6/9, the former referred to the number of successful rats in each group and the latter referred to total numbers of preparations in this group); (2) Met group (n = 6/11). The Met group was given metformin (50 mg/kg) twice a day from the first day after operation, while the Control group was given the same volume of 0.9% saline.

### Image acquisition, reconstruction and analysis

Rats were anesthetized with isoflurane. Serial gated ^18^F-FDG (40Mbq) micro-PET/CT imaging was performed at different time points (day 1st, day 7th, day 14th and day 30th after operation). Thickness, matrix, acquisition time and energy window of PET scan were 0.78 mm, 128 mm × 128 mm, 20 min and 350–650 keV respectively. Voltage, current, thickness and acquisition time of CT scanning were 80 kV, 500 µA, 0.1 mm and 10 min respectively. After scanning, the iterative reconstruction (4 iterations and 8 subsets) was performed by using OSEM 3D. Volumes of interest (VOIs) were drawn to identify different myocardium regions (ischemia center, peri-ischemia area and remote area). We defined apex as ischemia center, the border as peri-ischemia area and the posterior wall as remote area. Here, the target/background ratio (TBR) referred to the center/remote ratio. Standardized uptake values (SUVs) (SUV_mean_ and SUV_max_) were analyzed to evaluate the FDG uptake activity. In addition, the LV end-diastolic volume (EDV), end-systolic volume (ESV) and LV ejection fraction (LVEF) were obtained.

### Histological studies

All rats were sacrificed on the 30th day after PET/CT image acquisition. Thoracic cavity was cut open along the midsternal line. The heart was collected and washed with 0.9% sodium chloride solution. The atrium was cut along the coronal sulcus, and then the interventricular septum and right ventricle were cut along the septum to obtain the left ventricular myocardium. After being fixed with 4% paraformaldehyde and embedded in paraffin, the slices were continuously cut with a thickness of 5 µm. One out of every 5 sections were stained with routine HE.

### Statistical analysis

Statistical analyses were carried out by SPSS 26.0 (IBM SPSS® Statistics) software. The measured data conforming to normal distribution were expressed as mean (M) ± standard deviation (SD). Counting data and classification variables were given as frequency or percentage (%). All the results were calculated three times. One-way ANOVA was used for comparison between different groups, and non-parametric T test was used for comparison at different time points within the same group. Pearson's correlation analysis was used to analyze the relationship between TBR and cardiac function parameters. For all the tests, *p* < 0.05 was considered to be statistically significant.

## Results

### ^***18***^***F-FDG micro-PET/CT quantitative analysis***

**Fig. 1:**
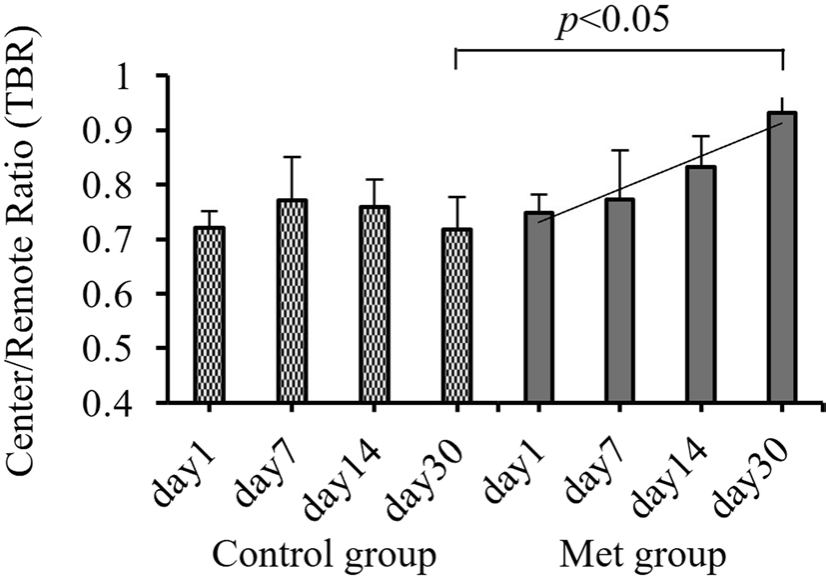
Change of TBR (Center/Remote ratio) at different time points in Control Group and Met Group

SUV_max_ value in different areas, ratio of SUV_max_ in central/remote area (TBR) and left ventricular function characteristics were summarized in Table 1.TBR and EDV showed statistical differences between two groups, while no significant differences were found in other parameters in the table. Especially in Met group, the TBR gradually increased with time, but there was no obvious changes trend at each time point in Control group. On day 30th, the TBR in Met group was significantly higher than that OF the Control group [(0.88 ± 0.06) vs. (0.72 ± 0.09), *p* < 0.05] (Fig. 1).

**Table 1:** SUV_max_ values and LV function characteristics at different time points in Metformin group (n = 6) and Control group (n = 6)

Indicators	Groups	Day 1st	Day 7th	Day 14th	Day 30th
Ischemia center (mm^3^)	Met	2.02 ± 0.40	1.80 ± 1.16	2.31 ± 0.84	1.91 ± 0.18
	Con	3.75 ± 2.33	3.20 ± 0.42	2.33 ± 0.17	3.22 ± 0.21
Peri-ischemia (mm^3^)	Met	2.61 ± 0.33	2.51 ± 1.34	2.70 ± 0.68	2.15 ± 0.94
	Con	4.32 ± 1.33	3.91 ± 0.98	2.85 ± 0.38	4.25 ± 0.98
Remote Area (mm^3^)	Met	2.41 ± 0.80	2.14 ± 0.52	2.82 ± 0.88	2.33 ± 0.44
	Con	4.15 ± 0.99	3.60 ± 0.17	2.80 ± 0.14	4.85 ± 0.41
Center/Remote ratio	Met	0.71 ± 0.16	0.73 ± 0.05	0.78 ± 0.13	0.88 ± 0.06^#^
	Con	0.72 ± 0.18*	0.77 ± 0.14*	0.76 ± 0.17*	0.72 ± 0.09^#^*
ESV (mm^3^)	Met	88.10 ± 15.58	60.10 ± 34.31	92.66 ± 36.50	68.00 ± 8.48
	Con	80.50 ± 2.21	68.20 ± 24.55	99.12 ± 30.40	75.41 ± 6.23
EDV (mm^3^)	Met	254.20 ± 70.19	291.61 ± 65.58	329.67 ± 74.60	358.21 ± 22.62^#^
	Con	217.12 ± 19.79*	270.92 ± 26.61*	349.67 ± 46.66*	407.53 ± 29.91^#^*
LVEF (%)	Met	70.80 ± 2.24	79.59 ± 3.42	76.22 ± 3.12	81.01 ± 1.42
	Con	67.85 ± 2.28	78.41 ± 2.45	73.09 ± 2.99	83.58 ± 1.12

Data are presented as mean ± SD or percentage (%) of subjects

*ESV* end-systolic volume, *EDV* end-diastolic volume, *LVEF* left ventricular ejection fraction

^#^Comparison between Met group and Control group, *p* < 0.05

*Correlation analysis between Center / Remote ratio and EDV, *p* < 0.05

### Met attenuated IR-induced LV dysfunction

**Fig. 2:**
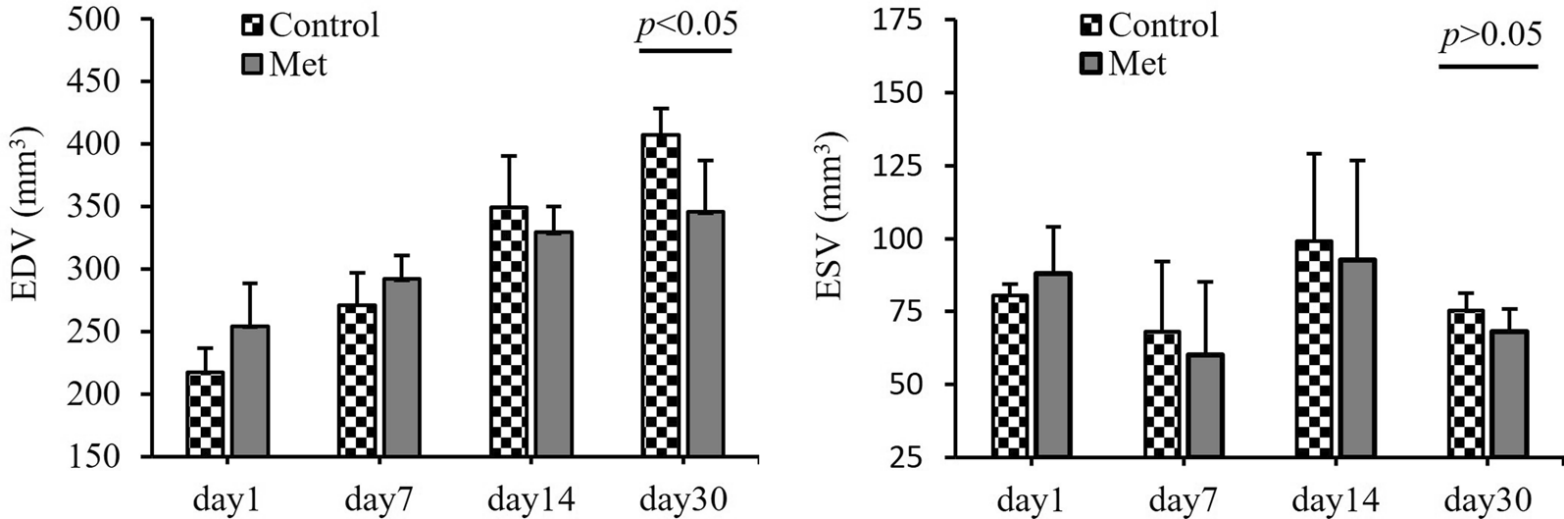
Change of LV ESV and EDV at different time points in Control group and Met group. *ESV* end-systolic volume, *EDV* end-diastolic volume

No significant differences in LV ESV values were found between two groups at different time points. However, from day 1st to day 30th after operation, the LV EDV values gradually increased, which was more significant in the Control group [(358.21 ± 22.62) vs. (407.53 ± 29.91) mm^3^, *p* < 0.05]. This indicated that Met could slow down the enlargement of LV secondary to I/R injury (Fig. 2).

### Met reduced heart infarct severity and LV size

**Fig. 3:**
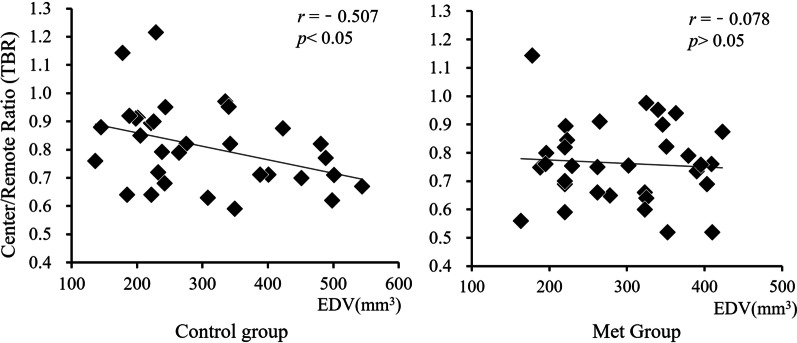
Correlation analysis between EDV and TBR (Center/Remote ratio). EDV, end-diastolic volume

**Fig. 4:**
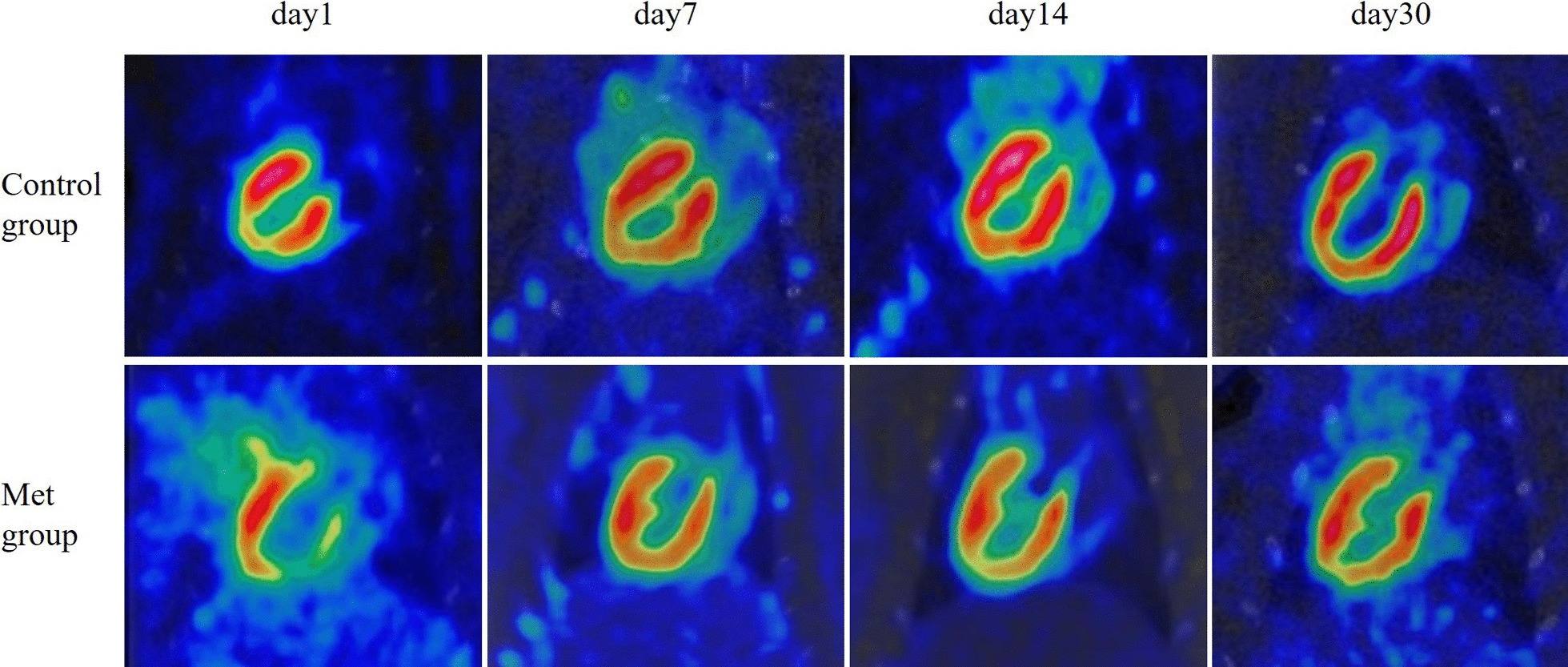
Representative PET/CT images of rat hearts at different time points from ischemia/reperfusion injury model

The TBR and LV EDV were negatively correlated in Control group (r = − 0.507, *p* < 0.05), while there was no significant correlation in Met group (r = − 0.078, *p* > 0.05) (Fig. 3). This demonstrated that as the enlargement of the cardiac chamber, myocardial damage in the ischemic central area is gradually aggravated without Met intervention. Imaging showed that LV size in the Control group increased gradually with time. However, there was no significant changes at different time points in Met group. On the day 30th after operation, the LV size in Met group was significantly smaller than that of the Control group (Fig. 4).

### Histological studies

**Fig. 5:**
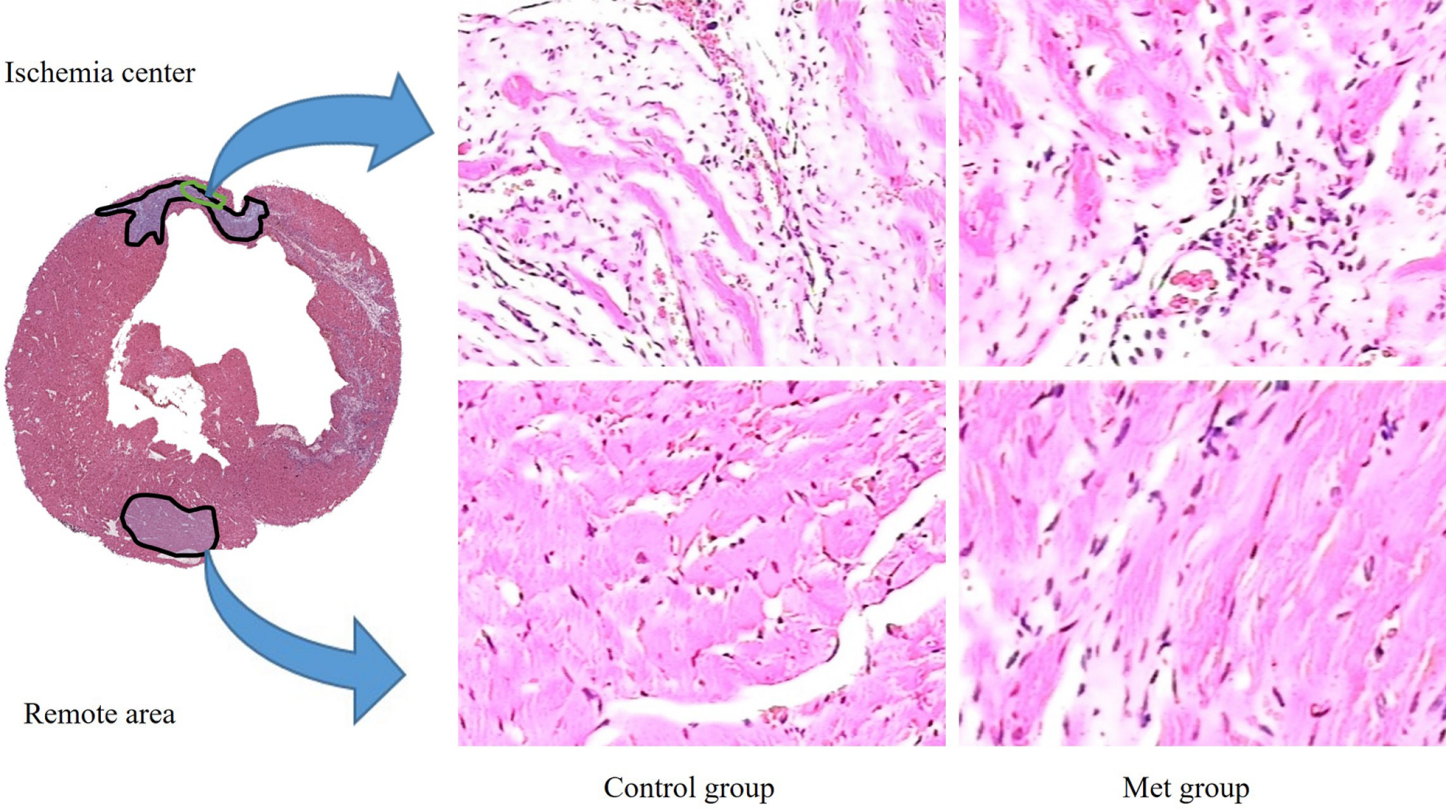
Representative HE staining of myocardial tissues in Control group and Met group after day 30th imaging acquisition in the ischemia center (×10) and remote area (×20)

HE staining of myocardial tissue sections in ischemia center area of rats in both groups showed that myocardial cells were swollen, with uneven distribution of cytoplasm, granular agglutination, eosinophilia and blurred or disappeared stripes (Fig. 5). It was confirmed that the myocardial infarction and fibrosis in the ischemic central area of the Control group were more serious than those in the Met group. Biopsies from two groups in remote areas showed normal myocardial tissue with clear striations and intact cell membranes and nuclei.

## Discussion

Metformin, as a safe, effective and relatively low-cost drug, was recommended as the first choice in the treatment of T2DM complicated with heart failure by the clinical guidelines of American Diabetes Association in 2014. Interestingly, it has also been documented for cardioprotective effects in animal researches and clinical trials [24, 25]. However, the underlying mechanism is still controversial. A recent meta-analysis [26] including 27 in vitro (myocardium and heart) and in vivo studies (animal experiments and clinical trials) found that Met treatment in I/R models could reduce the area of myocardial ischemia and infarction, reduce the degree of ventricular remodeling (EDV and ESV), and improve cardiac function and LVEF. The cardioprotective effect has also been confirmed in many models of acute cardiac injury. Solskov et al. [27] reported that Met exerted a protective effect on rat heart subjected to cardiac I/R injury in vitro when administered before a coronary occlusion was inflicted. Another study [28] confirmed the cardioprotective effect of Met in mice in vivo, also when administered during the reperfusion stage. However, only pathologic and immunohistochemical methods were used in the acute stage or at the end of treatment. There were no reports of dynamic, systematic and continuous in vivo imaging studies.

^18^F-FDG PET/CT imaging is internationally recognized as the "gold standard" for evaluating myocardium viability. The information such as myocardial perfusion, myocardial metabolism, myocardial cell activity, local systolic function, left ventricular function and ventricular remodeling can be obtained at the same time [29]. Therefore, in this study, ^18^F-FDG PET/CT technology was used to explore the effect and potential value of Met intervention on MIRI in rats from the perspective of molecular imaging. Our current research was unique in that a serial gated myocardial metabolism imaging by micro-PET/CT was performed in rats I/R injury model in acute phase (day 1st, day 7th), subacute phase (day 14th) and chronic phase (day 30th) respectively after modeling. The effects of Met on heart were evaluated dynamically, including the uptake (SUV_max_) in different myocardium regions (ischemia center, peri-ischemia area and remote area), and left ventricular function. TBR value of Met group gradually increased, and was significantly higher than that of Control group on day 30th. These results showed that Met improved FDG uptake in myocardium in ischemic center and reduced the severity of I/R injury with time. From day 1st to day 30th after operation, the LV EDV values in the Control group gradually increased. On the day 30th, it was more significant than that in Met group, which indicated that Met may prevent or delay the progress of LV remodeling secondary to I/R injury. The key finding was that after 1 month treatment, Met intervention not only reduced the severity of I/R injury but also delayed the ventricular remodeling. For the first time, in vivo molecular imaging proved that Met treatment can improve the glucose metabolic activity of ischemic myocardium, delay the occurrence and development of ventricular remodeling. It was also proved that Met treatment had myocardial protective effect on ischemic myocardium at molecular level.

The identification of inflammation and viable myocardium is a common problem [30]. Earlier studies have already demonstrated the metabolic changes in myocardium, skeletal muscle, and brain in mouse model under different conditions, i.e., fasted /non-fasted. In the non-fasted state, the uptake of imaging agent by cardiomyocytes was higher; while in the fasted state, the uptake of imaging agent by cardiomyocytes was lower, and the FDG uptake at this time mainly reflected inflammatory uptake. In this study, rats were fed with a normal diet, which mainly reflected the viable myocardium [31] In the follow-up studies, ammonia PET myocardial perfusion imaging would be added for integrated multimodal acquisition.

There are also some deficiencies in this study. First of all, the end time of Met intervention treatment was only 30 days. Severe I/R injury may not fully develop in such a short period of time, which could explain why the LVEF did not change significantly with the passage of time in this study. Here, research with longer intervention time and larger sample size is needed in the future. Secondly, only one treatment regimen was adopted at the beginning of reperfusion, which might be improved with a more complete regimen, including Met intervention before reperfusion, at the beginning of reperfusion and later after reperfusion. Finally, only a single dose (50 mg/kg twice a day) was used, which resulted in a lack of comparative analysis of high and low dose to evaluate the cardioprotective efficacy with different dosages. These deficiencies will be improved in further research.

## Conclusions

In summary, our study demonstrated that the Met could attenuate the severity of I/R injury on myocardium and delay or prevent the progress of LV remodeling. The cardioprotective effect could be dynamically evaluated by ^18^F-FDG micro-PET/CT imaging. These findings underscore the potential beneficial effects of Met in MIRI and provide further evidence that Met should be assessed prospectively in a long-term study. In addition, further investigations are needed to evaluate the effects of different dose of Met on MIRI.

## Data Availability

The datasets used and analyzed during the current study are available from the corresponding author on reasonable request.

## Abbreviations

Met: Metformin
MIRI: Myocardial ischemia–reperfusion injury
LAD: Left anterior descending
VOIs: Volumes of interest
SUVs: Standardized uptake values
LV: Left ventricular
EDV: End-diastolic volume
ESV: End-systolic volume
LVEF: Left ventricular ejection fraction
MI: Myocardial infarction
PCI: Percutaneous coronary intervention
T2DM: Type 2 diabetes mellitus
UKPDS: United Kingdom Prospective Diabetes Study
PET/CT: Positron emission tomography/computed tomography
TBR: Central/remote area

## References

[1] Virani SS, Alonso A, Aparicio HJ, Benjamin EJ, Bittencourt MS, Callaway CW (2021). Heart disease and stroke statistics-2021 update: a report from the American heart association. Circulation.

[2] Giblett JP, West NEJ, Hoole SP (2014). Cardioprotection for percutaneous coronary intervention-reperfusion quality as well as quantity. Int J Cardiol.

[3] Lattuca B, Kerneis M, Saib A, Nguyen LS, Payot L, Barthélemy O (2019). On- versus off-hours presentation and mortality of ST-segment elevation myocardial infarction patients treated with primary percutaneous coronary intervention. JACC Cardiovasc Interv.

[4] Silvis MJM, Kaffka Genaamd Dengler SE, Odille CA, Mishra M, van der Kaaij NP, Doevendans PA (2020). Damage-associated molecular patterns in myocardial infarction and heart transplantation: the road to translational success. Front Immunol.

[5] Okada Y, Numata T, Sato-Numata K, Sabirov RZ, Liu H, Mori SI (2019). Roles of volume-regulatory anion channels, VSOR and Maxi-Cl, in apoptosis, cisplatin resistance, necrosis, ischemic cell death, stroke and myocardial infarction. Curr Top Membr.

[6] Comità S, Femmino S, Thairi C, Alloatti G, Boengler K, Pagliaro P (2021). Regulation of STAT3 and its role in cardioprotection by conditioning: focus on non-genomic roles targeting mitochondrial function. Basic Res Cardiol.

[7] Zhang MW, Wang XH, Shi J, Yu JG (2021). Sinomenine in cardio-cerebrovascular diseases: potential therapeutic effects and pharmacological evidences. Front Cardiovasc Med.

[8] Hummitzsch L, Zitta K, Fritze L, Monnens J, Vollertsen P, Lindner M (2021). Effects of remote ischemic preconditioning (RIPC) and chronic remote ischemic preconditioning (cRIPC) on levels of plasma cytokines, cell surface characteristics of monocytes and in-vitro angiogenesis: a pilot study. Basic Res Cardiol.

[9] Turer AT, Hill JA (2010). Pathogenesis of myocardial ischemia-reperfusion injury and rationale for therapy. Am J Cardiol.

[10] Ekeløf S, Jensen SE, Rosenberg J, Gögenur I (2014). Reduced oxidative stress in STEMI patients treated by primary percutaneous coronary intervention and with antioxidant therapy: a systematic review. Cardiovasc Drug Ther.

[11] Davidson SM, Ferdinandy P, Andreadou I, Bøtker HE, Heusch G, Ibáñez B (2019). Multitarget strategies to reduce myocardial ischemia/reperfusion injury: JACC review topic of the week. J Am Coll Cardiol.

[12] Li C, Liu Z, Shi R (2021). A bibliometric analysis of 14,822 researches on myocardial reperfusion injury by machine learning. Int J Env Res Pub He.

[13] Driver C, Bamitale KDS, Kazi A, Olla M, Nyane NA, Owira PMO (2018). Cardioprotective effects of metformin. J Cardiovasc Pharm.

[14] Basnet S, Kozikowski A, Makaryus AN, Pekmezaris R, Zeltser R, Akerman M (2015). Metformin and myocardial injury in patients with diabetes and ST-segment elevation myocardial infarction: a propensity score matched analysis. J Am Heart Assoc.

[15] Nesti L, Natali A (2017). Metformin effects on the heart and the cardiovascular system: a review of experimental and clinical data. Nutr Metab Cardiovas.

[16] Higgins L, Palee S, Chattipakorn SC, Chattipakorn N (2019). Effects of metformin on the heart with ischaemia-reperfusion injury: evidence of its benefits from in vitro, in vivo and clinical reports. Eur J Pharmacol.

[17] Group UPDS. Effect of intensive blood-glucose control with metformin on complications in overweight patients with type 2 diabetes (UKPDS 34). UK Prospective Diabetes Study (UKPDS) Group. Lancet (London, England). 1998;352:854–65. 9742977

[18] Wang XF, Zhang JY, Li L, Zhao XY, Tao HL, Zhang L (2011). Metformin improves cardiac function in rats via activation of AMP-activated protein kinase. Clin Exp Pharmacol P.

[19] Palee S, Higgins L, Leech T, Chattipakorn SC, Chattipakorn N (2020). Acute metformin treatment provides cardioprotection via improved mitochondrial function in cardiac ischemia / reperfusion injury. Biomed Pharmacother.

[20] Zhang J, Huang L, Shi X, Yang L, Hua F, Ma J (2020). Metformin protects against myocardial ischemia-reperfusion injury and cell pyroptosis via AMPK/NLRP3 inflammasome pathway. Aging.

[21] Ferreira AG, Nunes da Silva T, Alegria S, Cordeiro MC, Portugal J. Paraganglioma presenting as stress cardiomyopathy: case report and literature review. Endocrinol Diabetes Metab Case Rep 2019;2019(1). 10.1530/EDM-19-0017 PMC6477651 30991354

[22] Bucerius J, Duivenvoorden R, Mani V, Moncrieff C, Rudd JHF, Calcagno C (2011). Prevalence and risk factors of carotid vessel wall inflammation in coronary artery disease patients: FDG-PET and CT imaging study. JACC-Cardiovasc Imag.

[23] Srivatsava MK, Indirani M, Sathyamurthy I, Sengottuvelu G, Jain AS, Shelley S (2016). Role of PET-CT in the assessment of myocardial viability in patients with left ventricular dysfunction. Indian Heart J.

[24] Zhao M, Xia LU, Tian Y, Meng J, Xie X, Wang H (2019). The long-term prognostic value of left ventricular dyssynchrony assessed by phase analysis of ^99m^Tc-MIBI gated SPECT in patients with left ventricular aneurysm: a comparative study of medical and surgical treatment. J Nucl Med.

[25] Wei H, Wang W, Yang Y, Nan J, Xiang L, Hacker M (2016). Bone marrow and splenic metabolic activity after acute coronary syndrome (ACS) was detected by ^18^F-FDG PET imaging. J Nucl Med.

[26] Hesen NA, Riksen NP, Aalders B, Brouwer MA, Ritskes-Hoitinga M, El Messaoudi S (2017). A systematic review and meta-analysis of the protective effects of metformin in experimental myocardial infarction. PLoS ONE.

[27] Solskov L, Løfgren B, Kristiansen SB, Jessen N, Pold R, Nielsen TT (2010). Metformin induces cardioprotection against ischaemia/reperfusion injury in the rat heart 24 hours after administration. Basic Clin Pharmacol.

[28] Gundewar S, Calvert JW, Jha S, Toedt-Pingel I, Lefer DJ (2009). Activation of AMP-activated protein kinase by metformin improves left ventricular function and survival in heart failure. Circ Res.

[29] Lu Y, Tian Y, Mou TT, Tian J, Zhou YH, Wen WW (2021). Effects of remote ischemic conditioning in pigs with acute myocardial infarction evaluated by serially gated ^99^Tc^m^-MIBI SPECT/CT and ^18^F-FDG PET/CT. Chin J Nucl Med Mol Imaging.

[30] Rischpler C, Dirschinger RJ, Nekolla SG, Kossmann H, Nicolosi S, Hanus F (2016). Prospective evaluation of 18F-Fluorodeoxyglucose uptake in postischemic myocardium by simultaneous positron emission tomography/magnetic resonance imaging as a prognostic marker of functional outcome. Circ Cardiovasc Imag.

[31] Michael K, David S, Koon-Pong W (2011). Influence of dietary state and insulin on myocardial, skeletal muscle and brain [18F]-fluorodeoxyglucose kinetics in mice. Ejnmmi Res.

